# Details in the evaluation of circular RNA detection tools: Reply to Chen and Chuang

**DOI:** 10.1371/journal.pcbi.1006916

**Published:** 2019-04-25

**Authors:** Xiangxiang Zeng, Wei Lin, Maozu Guo, Quan Zou

**Affiliations:** 1 Shenzhen Research Institute of Xiamen University, Shenzhen, China; 2 School of Information Science and Engineering, Xiamen University, Xiamen, China; 3 School of Electrical and Information Engineering, Beijing University of Civil Engineering and Architecture, Beijing, China; 4 Institute of Fundamental and Frontier Sciences, University of Electronic Science and Technology of China, Chengdu, China; University of Canterbury, NEW ZEALAND

Chia-Ying Chen and Trees-Juen Chuang (referred as CYC & TJC below) recently submitted their comment [1] on our previous paper [2]. In their paper, they scrutinized the CircBase [3] candidates that we used and pointed out several weak points of our paper. In summary, they suggested that the positive dataset we derived from CircBase required further evaluation. They also indicated that using all of these candidates as our dataset was not appropriate. They further suggested that three main confounding factors may affect our assessment of circRNA detection tools and that their performances should be re-evaluated.

Before we begin to discuss their comment, we will briefly introduce the positive dataset we used. First, as stated in our previous paper, the 14,689 candidates detected in HeLa cells were downloaded from CircBase and reported by the study of Salzman *et al*. [4]. These candidates were not identified with the use of find_circ [5] tool. As described in the study of Salzman *et al*. [4], all UCSC annotated exons in scrambled order were used to construct a custom database and identify circRNA candidates. Second, in our positive dataset, constant coverage of 10× for the intervening sequence and a minimum of two read pairs (paired-end simulated reads) to cross the back-spliced junction sites were generated for each candidate.

Now, we will discuss the three confounding factors they listed in their paper.

First, they suggested to remove 1046 candidates with unannotated exon boundaries from the positive dataset, especially candidates without canonical splice signals, such as GT-AG, GC-AG, or AT-AC, for the junctions. As mentioned above, CircBase-deposited circRNA candidates that we used were identified by Salzman *et al*. [4]; the candidates identified by their method should all match the exon boundaries. The discrepancies may be caused by inconsistent gene annotation files used. Salzman *et al*. [4] used UCSC known genes [6], whereas CYC & TJC used NCBI RefSeq-identified mRNA annotation files. We manually checked several candidates marked with “junctions with unannotated exon boundaries” in CYC & TJC’s Supplemental Dataset S1. The junction sites of these candidates were annotated as exon boundaries in UCSC known genes annotation file (http://hgdownload.soe.ucsc.edu/goldenPath/hg19/database/knownGene.txt.gz). Thus, detection of circRNAs with annotated exon boundaries relies on the gene annotation files used, and novel candidates may be missed because of the incompleteness of the current database [7]. For example, Szabo *et al*. [7] reinforced an annotation-based algorithm with a *de novo* module and discovered a validated circRNA from the not-fully-annotated *RMST* gene and several U12 circRNAs produced from unannotated boundaries. Such case was also demonstrated by Xiao-Ou Zhang *et al*. [8]. They detected thousands of novel exons (non-RefSeq, non-Ensembl, or non-UCSC known genes) in circRNAs by using an updated CIRCexplorere2 tool, and several of them were confirmed by Northern blot analysis and Sanger sequencing after RT-PCR [8]. Other examples were shown by Salzman *et al*. [4], they found several noncoding RNA genes expressed circular isoforms in mouse and human [4]. Gao *et al*. also provided evidence of intronic or intergenic circRNAs [9]. Moreover, the well-known *CDR1as* [5, 10] is an intergenic circRNA by definition. To study the mechanism of circularization, Starke *et al*. observed that both canonical splice sites are essential; however, they also cannot rule out the potential use of cryptic sites for circularization [11]. Their experimental data showed that when the normal 5′ or 3′ splice site was mutated, circRNAs can also be formed with the use of cryptic, noncanonical 5′ and 3′ splice sites [11]. Given the above-mentioned evidence, excluding candidates with unannotated exon boundaries or without canonical splicing sites is subject to discussion.

Second, they suggested the removal of 2316 candidates, of which the concatenated exon sequences flanking back-spliced junction sites exhibited ambiguous alignments. We checked these candidates on HeLa and Hs68 samples. As shown in Table 1, we found that some of them were not depleted (≥ onefold enrichment) or even significantly enriched (≥ fivefold enrichment) after RNase R treatment. (A Detailed discussion on two examples can be referred to Section I of the Supplementary File.) Therefore, suggesting that all of the candidates with ambiguous alignments are false calls and should be excluded from the analysis is inappropriate. However, sequencing reads produced from these candidates may result in multiple hits due to their ambiguous alignments, and it’s important to take into account of factors, such as sequencing base quality, alignment mismatches, minimum number of bases overhang both sides of the junction sites, and mapping uniqueness of the supporting back-spliced junction reads [7].

**Table 1:** ‘2316 ambiguous CircBase circRNAs’ on HeLa and Hs68 samples.

Dataset	HeLa						Hs68					
Tools	RNaseR-	RNaseR+	Not depleted	Percent (%)	Enriched	Percent (%)	RNaseR-	RNaseR+	Not depleted	Percent (%)	Enriched	Percent (%)
CF	110	168	79	71.82	24	**21.82**	102	407	85	83.33	59	57.84
CE	110	167	79	71.82	24	**21.82**	103	407	86	83.50	60	58.25
CIRI	148	**217**	111	**75.00**	**27**	18.24	126	390	112	88.89	81	**64.29**
DCC	96	137	64	66.67	17	17.71	91	330	81	**89.01**	53	58.24
FC	82	91	41	50.00	11	13.41	52	227	40	76.92	32	61.54
KNIFE	170	199	111	65.29	25	14.71	131	395	109	83.21	73	55.73
MS	88	122	61	69.32	11	12.50	70	249	61	87.14	42	60.00
NCLS	34	37	19	55.88	3	8.82	23	100	16	69.57	11	47.83
PF	**186**	206	**114**	61.29	26	13.98	**141**	**449**	**118**	83.69	**84**	59.57
SG	178	213	105	58.99	23	12.92	137	366	80	58.39	56	40.88
UB	55	63	20	36.36	3	5.45	29	64	5	17.24	4	13.79

Note: Candidates with ≥ 2 supporting back-spliced junction reads were used in the analysis, and the number of supporting reads was normalized with sequencing depth before fold change calculation. After RNase R treatment, detected candidates with ≥ onefold enrichment was defined as ‘Not depleted’, while candidates with ≥ fivefold enrichment was regarded as ‘Enriched’. CF: circRNA_finder; CE: CIRCexplorer; FC: find_circ; MS: MapSplice; SG: Segemehl; NCLS: NCLScan; PF: PTESFinder; UB: UROBORUS.

Third, they suggested that “unqualified reads” with ambiguous alignments and different supporting read counting methods of the tools affected our reported results. First, we would like to clarify that the result of CIRI, MapSplice, and find_circ that we provided in our previous paper [2] only included candidates with ≥ 2 supporting back-spliced junction reads because of the limited output with default parameter setting of the three tools. Thus, no circRNAs with one supporting reads for these tools are included in Fig 3B of CYC & TJC’s comment paper. If candidates with one supporting reads were reported by the three tools, then the total number of CircBase circRNAs identified by all 11 tools is expected to be more than 3580 events (Fig 3B of CYC & TJC’s comment paper). As for “unqualified reads”, the 4 reads they listed in Fig 3C of their paper were back-spliced junction reads generated by CIRI-simulator [9] to support this circRNA. (A detailed discussion on two of these reads can be referred to Section II of the Supplementary File.) As for “different counting methods” used by different tools, it possibly affects the detection of circRNAs with small size. If the spliced length of the candidates is smaller than the insert size of the sequencing library, then both mates of the paired-end reads possibly cross the back-spliced junction sites. If both mates of the paired-end reads cross the back-spliced junction site, then this case is beneficial to all tools because of increased opportunities to detect the back-spliced junction event. For Fig 4 of our previous paper, by focusing our analysis on common candidates with spliced length exceeding the insert size of the sequencing library, we eliminated the influence of different counting methods. For Table 1 of our previous paper, we generated sufficient (≥ 2) back-spliced junction reads for each circRNA in the positive dataset. And it was a common practice to keep candidates with ≥ 2 supporting reads for further analysis [12] [5, 9] [13], while reliable methods to reduce false-positive circRNAs still remains to be developed. In summary, it’s feasible to assess the sensitivity of each tool by keeping candidates with ≥ 2 supporting reads (Table 1 & Fig 4 of our previous paper).

Finally, CYC & TJC emphasized that either RTase- and non-RTase-based experiments or at least two different types of RTase-based experiments should be conducted to validate the authenticity of the circRNA candidates. We believe that the origins (from different tissues/cell lines) of our collected circRNAs will not affect the fairness of our evaluation. However, we acknowledge that not all of the 282 circRNAs, which we compiled from 17 published studies, were validated using methods indicated by CYC & TJC, such circRNAs should be collected if possible.

In our previous paper [2], to evaluate the performance of 11 circRNA detection tools, we generated a synthetic positive dataset from 14,689 candidates deposited in CircBase [3] that were previously identified from HeLa cells by using an annotation-based method [4]. Although the authenticity of these candidates still remains to be verified, they should all match the exon boundaries annotated in UCSC knownGene database [6]. In CYC & TJC’s comment paper, they further scrutinized these candidates. After analysis, they suggested that three main confounding factors may compromise the fairness of our assessment. Consequently, they suggested the removal of candidates with unannotated exon boundaries, particularly those without canonical splice sites. In addition, they suggested to exclude candidates with ambiguous alignments. As discussed in a previous study [14] and also shown by our data, although these heuristic filtering steps can eliminate particular types of false positives, they may create blind spots and reduce sensitivity. Third, they suggested that our evaluation of the tools was affected by unqualified reads with ambiguous alignments and different supporting read-counting methods. However, all the unqualified reads listed in Fig 3C of the comment paper are back-spliced junction reads generated by CIRI-simulator [9]. The discrepancies may be caused by the failure of BLAT [15] to detect supporting reads of which only a small portion spans the back-spliced junction sites. In our previous paper, prior to further analysis, relevant steps were adopted to minimize the effect of different counting methods. In summary, CYC & TJC underlined several knowledge-based filtering steps and an experimental validation method to address the bioinformatic and experimental challenges in detecting circRNAs, but whether these heuristic filtering steps should be enforced still requires further discussion. Finally, we reanalyzed the positive and mixed datasets with their suggested removal of ‘uncertain circRNA candidates’. Data in Table 1 of our previous paper were updated as Table 2 below. In general, our previous conclusions drawn from these two datasets are robust to the change.

**Table 2:** Summary of accuracy measures on the positive and mixed datasets.

Datasets	Positive						Mixed					
Tools	#Detected	TP	S (%)	P (%)	F1	AUC	#Detected	TP	S (%)	P (%)	F1	AUC
CIRI	10714	10686	92.29	99.74	0.96	0.92	10850	10668	92.13	98.32	0.95	**0.92**
CF	8186	8109	70.03	99.06	0.82	0.70	8239	8109	70.03	98.42	0.82	0.70
DCC	7506	7460	64.43	99.39	0.78	0.64	7510	7460	64.43	99.33	0.78	0.64
FC	9085	9035	78.03	99.45	0.87	0.78	9795	9035	78.03	92.24	0.85	0.66
SG	11381	10677	92.21	93.81	0.93	0.89	12126	10308	89.02	85.01	0.87	0.84
CE	9970	9936	85.81	99.66	0.92	0.86	9972	9936	85.81	99.64	0.92	0.86
MS	8208	8168	70.54	99.51	0.83	0.70	8206	8159	70.46	99.43	0.82	0.70
UB	8434	7985	68.96	94.68	0.80	0.67	8517	7500	64.77	88.06	0.75	0.58
KNIFE	11406	11360	**98.11**	99.60	**0.99**	**0.98**	11819	11300	**97.59**	95.61	**0.97**	**0.92**
PF	10496	10458	90.32	99.64	0.95	0.90	10524	10465	90.38	99.44	0.95	0.90
NCLS	7218	7214	62.30	**99.94**	0.77	0.62	7220	7216	62.32	**99.94**	0.77	0.62

TP: true positives; S: sensitivity; P: precision; F1: F1 score; AUC: area under precision/recall curve; CF: circRNA_finder; CE: CIRCexplorer; FC: find_circ; MS: MapSplice; SG: Segemehl; NCLS: NCLScan; PF: PTESFinder; UB: UROBORUS. Note: there were a total of 11579 true positives in these two datasets. After we removed the ‘uncertain circRNA candidates’ listed in CYC & TJC’s comment paper, 3110 candidates instead of 3150 (probably a typo in their paper) were obtained after we merged the data from their DataSet S1 and Dataset S2 files.

## Supporting information

S1 File (I) Examples of not-depleted or even enriched “ambiguous CircBase circRNAs” after RNase R treatment. (II) Examples of back-spliced junction read pairs being mistaken as “unqualified reads”.
(DOCX)
