# Supplementary material for: Details in the evaluation of circular RNA detection tools: Reply to Chen and Chuang
Source: PLoS Comput Biol. 2019 Apr 25;15(4):e1006916. doi: 10.1371/journal.pcbi.1006916 (PMC6527241; doi:10.1371/journal.pcbi.1006916)
Supplement: S1 File — (I) Examples of not-depleted or even enriched “ambiguous CircBase circRNAs” after RNase R treatment. (II) Examples of back-spliced junction read pairs being mistaken as “unqualified reads”. (DOCX) [file pcbi.1006916.s001.docx]

(I) Examples of not-depleted or even enriched “ambiguous CircBase circRNAs” after RNase R treatment.

First, chrX:47705504|47755339. It was detected on HeLa samples by 8 out of the 11 tools (except NCLscan, Segmehl, and UROBORUS), except one of them showed it was not depleted (4.6-fold enrichment), the remaining 7 tools reported it was significant enriched after RNase R treatment. Meanwhile, this candidate was also detected by the same 8 tools on Hs68 samples, with all of them reporting more than 22-fold enrichment after RNase R treatment. Second, chr6:111208708|111211559. On HeLa samples, it was detected by 10 tools (except Segemehl), with 6 of them reporting significant enrichment, while the remaining 4 showed non-depletion after RNase R treatment. On Hs68 samples, 9 out of the 11 tools (except UROBORUS and Segemehl) detected and reported more than 15-fold enrichment after RNase R treatment.

(II) Examples of back-spliced junction read pairs being mistaken as “unqualified reads”.

First, the simulate:514081 read pair. As shown in Fig. 3C (bottom) of the comment paper, its read1 (the first 101 bp) can be perfectly mapped to chr9:131085302-131085402, and its last 76 bp of read2 (the second 101 bp) can be perfectly mapped to chr9:131088085-131088160. Yet, its read2 is a back-spliced junction read, with its first 25 bp being perfectly mapped to chr9:131085295-131085319. Second, the simulate:514082 read pair (Fig. 3C, middle). It is a more complicated read-pair, with both mates crossing the junction sites. The last 88 bp of its read1 can be perfectly mapped to chr9:131085295-131085382, while its first 13 bp can be perfectly mapped to chr9:131088148-131088160. Meanwhile, the last 96 bp of its read2 can be perfectly mapped to chr9:131088065-131088160, and its first 5 bp can also be perfectly mapped to chr9:131088319-131088323 despite being generated from chr9:131085295-131085299.
